# New Understanding of Meta-Topolin Riboside Metabolism in Micropropagated Woody Plants

**DOI:** 10.3390/plants13091281

**Published:** 2024-05-06

**Authors:** Maroua Grira, Els Prinsen, Stefaan Werbrouck

**Affiliations:** 1 Laboratory for Applied In Vitro Plant Biotechnology, Ghent University, Valentin Vaerwyckweg 1, 9000 Ghent, Belgium; 2 Integrated Molecular Plant Physiology Research, Department of Biology, University of Antwerp, Groenenborgerlaan 170, 2020 Antwerp, Belgium

**Keywords:** cytokinin metabolism, labeled meta-topolin riboside, *Handroanthus guayacan*, *Tabebuia rosea*, *Tectona grandis*

## Abstract

Topolin cytokinins have emerged as valuable tools in micropropagation. This study investigates the metabolism of meta-topolin riboside (mTR) in three distinct tree species: *Handroanthus guayacan* and *Tabebuia rosea* (*Bignoniaceae*), and *Tectona grandis* (*Lamiaceae*). Employing labeled N^15^ mTR, we unraveled the complex mechanisms underlying cytokinin homeostasis, identifying N9-glucosylation as the principal deactivation pathway. Our findings demonstrate a capacity in *T. rosea* and *H. guayacan* to reposition the hydroxyl group on the cytokinin molecule, a previously unexplored metabolic pathway. Notably, this study reveals remarkable interfamilial and interspecies differences in mTR metabolism, challenging established perspectives on the role of callus tissue in cytokinin storage. These insights not only illuminate the metabolic intricacies of mTR, a cytokinin with interesting applications in plant tissue culture, but also enhances our understanding of cytokinin dynamics in plant systems, thereby enriching the scientific discourse on plant physiology and cytokinin biology.

## 1. Introduction

The exploration of cytokinins (CKs) dates back to 1955 when Miller et al. [1] identified kinetin as the first cytokinin, recognizing its cell-division-promoting properties. Subsequently, in 1959, Okumura et al. [2] synthesized a spectrum of kinetin analogues and BA (benzyladenine) derivatives. Their activities remained elusive for nearly three decades until Kaminek et al. [3] unveiled the significant biological effects of topolins, in 1987. This revelation stimulated an intensified investigation into meta-topolin (mT), as reported by Nisler [4]. mT and its derivates have been demonstrated to be effective in resolving a range of in vitro issues, including root inhibition [5,6], early senescence [7,8], hyperhydricity [9,10], shoot tip necrosis [11], and decomposing chimeras [12]. Prior studies in *Aloe peglerae* [13], *Merwilla pluombea* [14], and *Pelargonium sidoide* [15] suggest that mTR (meta-topolin riboside) often outperforms mT in micropropagation efficacy. Despite this demonstrated superiority, mTR has not yet been widely adopted in micropropagation practices. 

The aim of this study was to investigate the metabolism of mTR using labeled N^15^ mTR and to comprehend the distribution of topolins and their conjugates across three different tree species. *Handroanthus guayacan* and *Tabebuia rosea* from the *Bignoniaceae* family were selected to identify potential similarities in metabolic pathways due to their phylogenetic proximity. *Tectona grandis* of the *Lamiaceae* family served as a comparative reference from a distinct tree family. *H. guayacan* is renowned as ‘Ipê’ and extensively harvested for its durable, high-quality wood, exhibiting fire resistance comparable to concrete and steel [16]. The family-related *T. rosea* shows promise in synthesizing silver nanoparticles with notable antioxidant and antibacterial properties [17]; it is commercialized for its high-quality wood and for the ecosystem services it provides [18]. *T. grandis* is widely utilized for its wood in constructing floors, cabinets, and boats. Traditionally employed for sculpting and everyday household items, a recent discovery highlighted the capacities of *T. grandis* leaves for wastewater treatment, dye removal, and biogas production [19].

## 2. Results

Based on their function, the quantified aromatic cytokinin and their conjugates were classified into four groups: N^15^ free bases, N^15^ ribosides, N^15^ O-glucosides, and N^15^ N-glucosides. N^15^ free bases include meta-topolin (N^15^ mT) and para topolin (N^15^ pT); N^15^ ribosides include meta-topolin riboside (N^15^ mTR) and para topolin riboside (N^15^ pTR); N^15^ O-glucosides include meta-topolin riboside O-glucoside (N^15^ mTROG), meta-topolin O-glucoside (N^15^ mTOG), para topolin O-glucoside (N^15^ pTOG) and ortho topolin O-glucoside (N^15^ oTOG), and N^15^ N-glucosides include meta-topolin-9-glucoside (N^15^ mT9G), ortho topolin-9-glucoside (N^15^ oT9G), and para topolin-9-glucoside (N^15^ pT9G).

**Figure 1:**
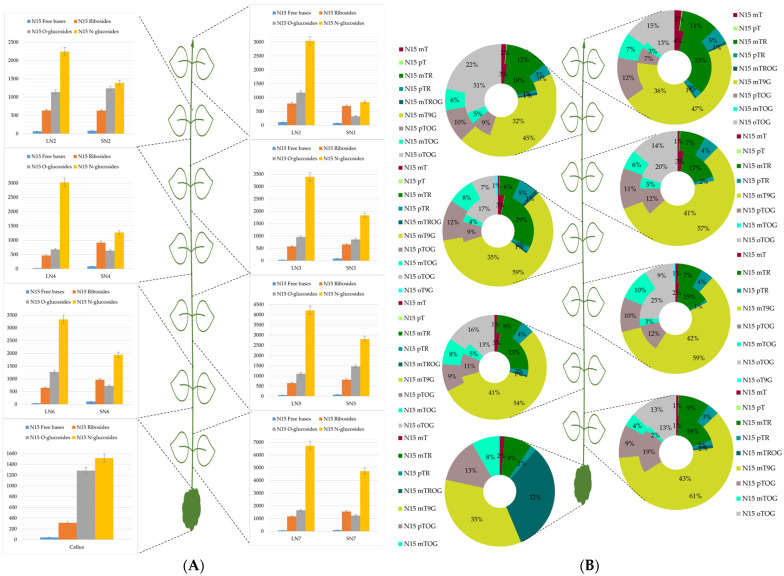
mTR and its metabolites in *Handroanthus guayacan*. (**A**): Concentrations expressed in picomoles per gram dry weight of the sum of all meta-topolin riboside conjugates (N^15^ free bases, N^15^ ribosides, N^15^ O-glucosides, and N^15^ N-glucosides) in leaves and stem of each node from top (N1) to the bottom (N7) and in callus. (**B**): Percentage distribution of all meta-topolin riboside conjugates (N^15^ mT, N^15^ pT, N^15^ mTR, N^15^ pTR, N^15^ mTROG, N^15^ mTOG, N^15^ pTOG, N^15^ oTOG, and N^15^ mT9G) in stems (inner cercle) and leaves (outer circle) of the doughnut from top (N1) to base (N7), and in callus. LN1: leaves of node 1, SN1: stem of node 1, LN2: leaves of node 2, SN2: stem of node 2, LN3: leaves of node 3, SN3: stem of node 3, LN4: leaves of node 4, SN4: stem of node 4, LN5: leaves of node 5, SN5: stem of node 5, LN6: leaves of node 6, SN6: stem of node 6, LN7: leaves of node 7, and SN7: stem of node 7.

**Figure 2:**
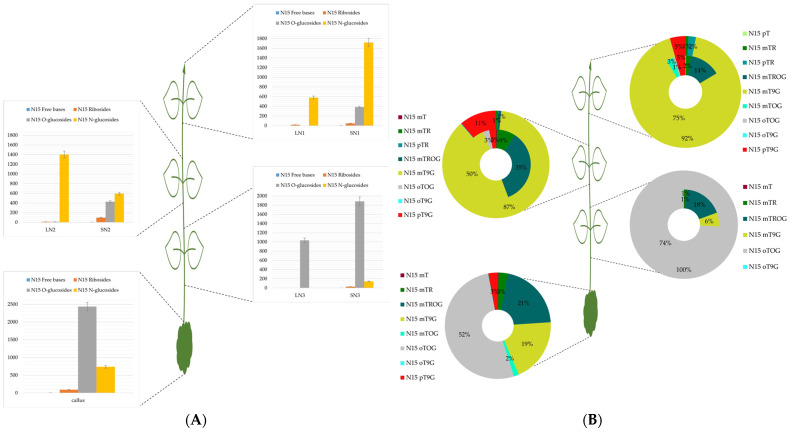
mTR and its metabolites in *Tabebuia rosea*. (**A**): Concentrations expressed in picomoles per gram dry weight of the sum of all meta-topolin riboside conjugates (N^15^ free bases, N^15^ ribosides, N^15^ O-glucosides, and N^15^ N-glucosides) in leaves and stems of each node from top (N1) to the bottom (N3) and in callus. (**B**): Percentage distribution of all meta-topolin riboside conjugates (N^15^ mT, N^15^ mTR, N^15^ pTR, N^15^ mTROG, N^15^ mTOG, N^15^ pTOG, N^15^ oTOG, N^15^ mT9G, and N^15^ oT9G) in stem (inner cercle) and leaves (outer circle) of the doughnut from top (N1) to base (N3), and in callus. LN1: leaves of node 1, SN1: stem of node 1, LN2: leaves of node 2, SN2: stem of node 2, LN3: leaves of node 3, and SN3: stem of node 3.

**Figure 3:**
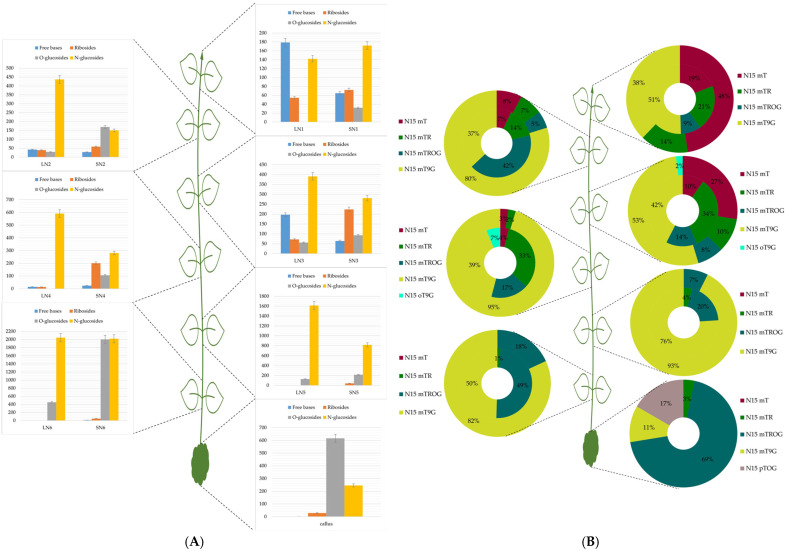
mTR and its metabolites in *Tectona grandis*. (**A**): Concentrations expressed in picomoles per gram dry weight of the sum of all meta-topolin riboside conjugates (N^15^ free bases, N^15^ ribosides, N^15^ O-glucosides, and N^15^ N-glucosides) in leaves and stem of each node from top (N1) to the bottom (N6) and in callus. (**B**): Percentage distribution of all meta-topolin riboside conjugates (N^15^ mT, N^15^ mTR, N^15^ mTROG, N^15^ pTOG, N^15^ mT9G, and N^15^ oT9G) in stems (inner cercle) and leaves (outer circle) of the doughnut from top (N1) to base (N6), and in callus. LN1: leaves of node 1, SN1: stem of node 1, LN2: leaves of node 2, SN2: stem of node 2, LN3: leaves of node 3, SN3: stem of node 3, LN4: leaves of node 4, SN4: stem of node 4, LN5: leaves of node 5, SN5: stem of node 5, LN6: leaves of node 6, and SN6: stem of node 6.

Figure 1A, Figure 2A, and Figure 3A represented the absolute concentrations of each group per leaf pair and per stem in *H. guayacan*, *T. rosea*, and *T. grandis*, respectively. And Figure 1B, Figure 2B, and Figure 3B represented the relative occurrence of mTR and all its metabolites per leaf pair and per stem in *H. guayacan*, *T. rosea*, and *T. grandis*, respectively. The legend of the doughnut chart contains all quantified conjugates (yet with a percentage <1%), but only those with percental values greater than 1% were displayed inside the chart. In the legend of the histogram, the letter L indicates the leaves, the letter N indicates the node, the letter S indicates the stem, and the number indicates the position of the node from the top (1) to the bottom.

### 2.1. Handroanthus Guayacan

An analysis of the labeled conjugate concentrations, as depicted in Figure 1A, revealed consistent N^15^ N-glucoside values across all leaves. These values ranged from 2.200 to 4.200 pmol/g DW, with a notable exception in the leaves of the basal node (N7), where the concentration of N^15^ N-glucosides was higher and reached nearly 7.000 pmol/g DW. A similar gradient was observed for N^15^ O-glucosides within all stems. Values ranged from nearly 800 to 3.000 pmol/g DW, again with the exception of the N7 stem, where the concentration of N^15^ N-glucosides peaked at nearly 5.000 pmol/g DW. N^15^ free bases remained below 50 pmol/g DW, while N^15^ ribosides did not exceed 1.000 pmol/g DW in stems and 600 pmol/g DW in leaves. In the callus, N^15^ N-glucosides and N^15^ O-glucosides exhibited comparable values of 1.500 and 1.300 pmol/g DW, respectively. Moreover, there, N^15^ ribosides and N^15^ free bases reached maximum concentrations of 350 and 50 pmol/g DW, respectively.

N^15^ mTR, the initial form which was added exogenously to the culture medium, was more abundant in the upper nodes, as detailed in Figure 1B. The leaves and stems of the upper first two nodes exhibited 11–12% and 33–18% respectively. Notably, N^15^ mTR was generally more concentrated in stems than in leaves. Similarly, the N^15^ mT distribution favored stems, with concentrations increasing towards the plant’s apex (N1). The peak values reached 4% in stems and 2% in leaves. The distribution of irreversible N9 conjugates indicates that N^15^ mT9G was predominant across all leaves, with a slightly increasing gradient from the top to the base of the plant, ranging from 47% to 61%. Similarly, in the stem, N^15^ mT9G was the most abundant conjugate, with values ranging from 36% and 43% from the top of the plant to the base. The reversible storage form, N^15^ mTROG, was found primarily in stems, except in leaves of N1 and N4. The relative amount of N^15^ mTROG did not surpass 1% in leaves and 2% in stems. The concentration of the para isomer (N^15^ pTR) was higher in leaves than in stems, with values between 3–5% in leaves and between 1–2% in stems. N^15^ mTOG was present in all leaves and stems, with slightly higher concentrations observed in leaves. Across plant nodes, N^15^ mTOG comprised between 6–10% of the leaf content and 2–5% of the stem content. The N^15^ pTOG isoform was found throughout the plant. In leaves, a subtle decreasing gradient toward the base was observed, with values ranging from 12 to 9%. Conversely, stems showed an increasing gradient from the apex to base (7–19%). However, the gradient was reversed and the concentration of N^15^ pTOG increased from 7% to 19% from the top to the base of the plant. On the other hand, N^15^ oTOG was measured in both the leaves and stems of all nodes of the plant, without a clear pattern. N^15^ oTOG in leaves ranged from 7–22%, and in stems from 13–31%. N^15^ oT9G was found only in leaves of N4 and N5 at concentrations not exceeding 1%. The concentrations of N^15^ oTOG in leaves ranged from 7–22% and in stems from 13–31%. N^15^ oT9G was only detected in leaves of nodes N4 and N5 at concentrations not exceeding 1%. An analysis of callus tissue showed that N^15^ mT9G and N^15^ mTROG were the primary metabolites (35% each). The remaining metabolites were N^15^ pTOG (13%), N^15^ mTOG (8%), N^15^ mT (2%), N^15^ mTR (8%), and N^15^ pTR (2%).

### 2.2. Tabebuia Rosea

Figure 2A reveals distinct patterns of the N^15^ mTR metabolite distribution across plant nodes. In N1 and N2, N^15^ N-glucosides significantly exceeded N^15^ O-glucosides in leaves, with concentrations increasing from 600 to 1.400 pmol/g DW. Stems exhibited an inverse pattern, where N^15^ N-glucosides decreased from 1.700 to 600 in N1 and N2. Intriguingly, this trend reversed in node 3 and callus tissue, where N^15^ O-glucosides were more abundant. Additionally, node 1 displayed a nearly threefold-higher concentration of N^15^ N-glucosides in stems compared to leaves. This relationship shifted in node 2, with leaves exhibiting more than double the N^15^ N-glucoside content of stems. N^15^ ribosides remained low throughout all nodes and the callus, with free bases present in negligible amounts.

In the apical node of the plant, the predominant conjugate was N^15^ mT9G, which constituted 92% in leaves and 75% in stems (Figure 2B). A similar trend persisted in N2, although with a slightly lower percentages of N^15^ mT9G: (87% in leaves, and 50% in stems). At node 3, a striking shift occurred: N^15^ oTOG became the exclusive conjugate in leaves (100%) and remained predominant in stems (74%). N^15^ mTROG was the second most abundant conjugate, detected exclusively in stems with proportions ranging from 14% to 35%. N^15^ pT9G was restricted to nodes 1 and 2, reaching 5% in both the leaf and stem tissues of node 1 and increasing to 11% (leaves) and 3% (stems) in node 2. N^15^ mTR was found in all nodes except the leaves of the third node, at 1% in leaves. Stem concentrations varied and were 2%, 9%, and 1% from the apex to base of the plant, respectively. N^15^ oTOG was detected throughout the plant. In stems of nodes 1 and 2, the proportions were 1% and 3%, respectively, increasing dramatically to 74% (stems) and 100% (leaves) in node 3. The callus showed a very distinct conjugate profile. oT9G was the dominant conjugate (52%), followed by N^15^ mTROG (21%) and N^15^ mT9G (19%). N^15^ pT9G, N^15^ mTR, and N^15^ mTOG were present in lower proportions (3% and 2%, respectively).

### 2.3. Tectona Grandis

Figure 3A shows that, in the leaves of the first three nodes, free bases showed higher concentrations compared to both N^15^ ribosides and N^15^ O-glucosides, particularly in the leaves of the N1 and N2, where N^15^ free bases significantly exceeded N^15^ ribosides (180 and nearly 60 pmol/g DW, respectively). With the exception of the leaves of N1 and the stem of N2, N^15^ N-glucosides emerged as the predominant group of labeled conjugates. The levels of N^15^ N-glucosides in leaves showed a progressive increase from the top to the base of the plant, ranging from nearly 140 pmol/g DW to nearly 2 nmol/g DW, respectively. Similarly, N^15^ N-glucosides in stems mirrored this increasing gradient observed in leaves, with values ranging from nearly 170 to nearly 2.000 pmol/g DW across the plant. N^15^ ribosides consistently showed higher concentrations than N^15^ free bases in the stems of all nodes.

Figure 3B shows that the predominant conjugate across the plant was N^15^ mT9G. In leaves, N^15^ mT9G showed a clear upward gradient toward the base of the plant, presenting a percentage varying between 38 and 95%, respectively. A parallel trend was observed in stems, where percentages ranged from 37% to 76% in favor of the base of the plant. Moreover, N^15^ mT was quantified in all nodes but only in the four initial nodes did its percentages exceed 1%. A clear increasing gradient for N^15^ mT was observed toward the top of the plant, with peak levels quantified in the leaves and stems of the apical shoot meristem (48% and 19%, respectively) and the lowest levels at the fourth node (3% and 4% in leaves and stem, respectively). The supplemented labeled N^15^ mTR was quantified in almost all nodes, but no gradient was detectable. The percentages exhibited slight variations along the plant, with a peak of 34% in the stems of N3 and 14% in the leaves of N1. However, the storage form (N^15^ mTROG) was quantified at higher levels compared to N^15^ mTR. The measurements of N^15^ mTROG showed a clear decreasing gradient from the base of the plant to the top, with concentrations ranging from 49% to 9% in the stems. The concentrations of conjugates in the leaves mirrored this gradient, ranging from 5% at the second, to 18% at the seventh node. Notably, N^15^ mTROG was not quantified in the leaves of the first and fourth nodes. Surprisingly, N^15^ oT9G was exclusively quantified in the third and fourth nodes, with values of 2% and 7% in the leaves and stems, respectively. In the entire plant, N^15^ mT9G was the major conjugate except in the callus. In the callus, N^15^ mT9G represented only 11%, overshadowed by N^15^ mTROG with 69%. N^15^ mTR represented only 3%, but a new conjugate, which was not quantified in the leaves and stems of the plant, was present in the callus at 17%: it was N^15^ pTOG.

## 3. Discussion

Research efforts have primarily concentrated on the study of endogenous isoprenoid cytokinins (ISCKs), resulting in a limited understanding of endogenous aromatic cytokinins (ARCKs) in plants. Of particular note are mT and mTR, which have transformed micropropagation practices [20]. This study provides a unique perspective on the mechanisms by which woody plants manage exogenous cytokinin applications, often used in high concentrations within tissue culture media to induce branching. Through the meticulous analysis of leaves and stems individually, coupled with the use of labeled N^15^ mTR, we present novel insights into the metabolism of this phytohormone. The distribution of ARCK conjugates exhibited distinct node-to-node variations. This observation emphasizes the intricate nature of mTR metabolism and suggests node-specific differences in the distribution of key conjugate groups.

**Figure 4:**
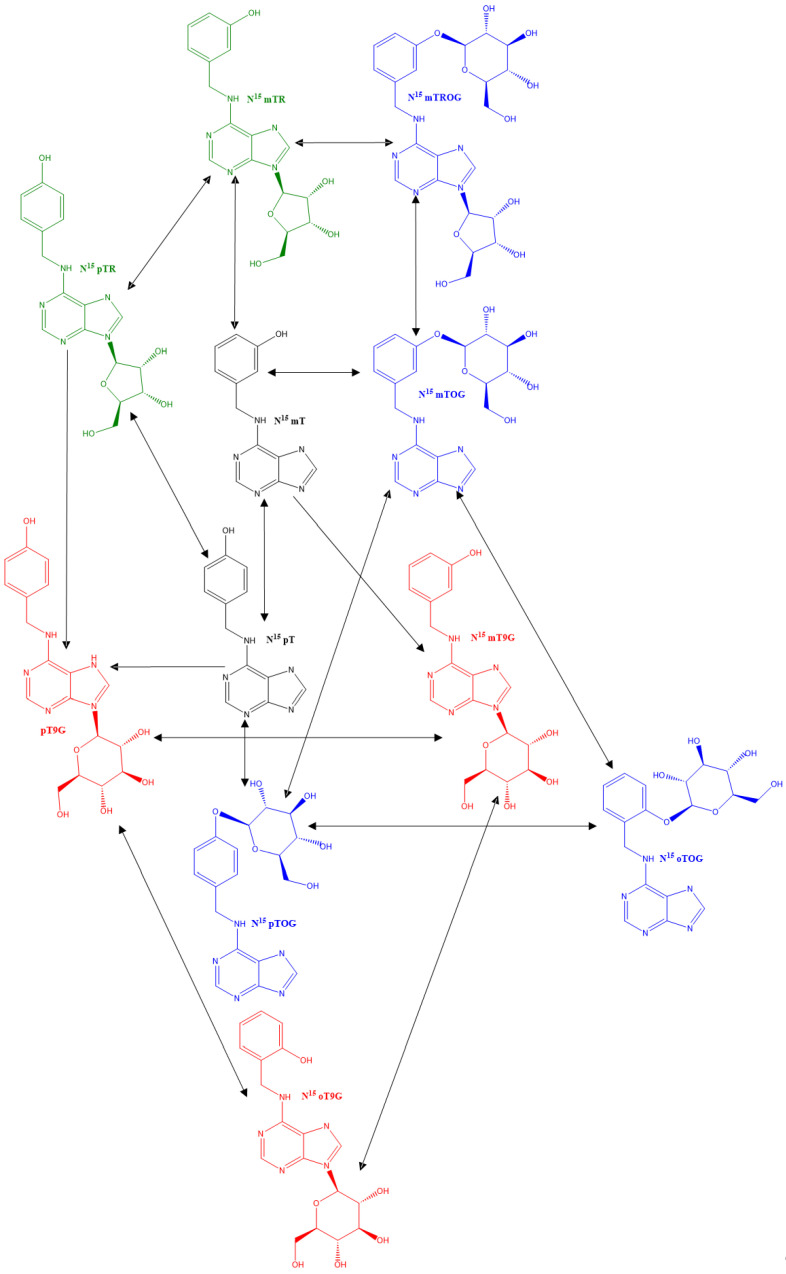
Interconversion of meta-topolin riboside derivatives in *Handroanthus guayacan, Tabebuia rosea*, and *Tectona grandis*. The metabolites include N^15^ free bases (black), N^15^ ribosides (green), N^15^ N-glucosides (red), and N^15^ O-glucosides (blue). The circle indicates the exogenously supplemented form of topolin.

As illustrated in Figure 4, our recovery of the free-base mT alongside inactive forms (pT, pTR) suggests a multifaceted regulatory network for cytokinin homeostasis in these plants. We demonstrate that both active (mT) and inactive (pT) cytokinins undergo N9-glucosylation on the adenine ring. Although N9-glucosyl conjugates are typically inactive due to hindered hydrolysis, they accumulate to high levels under normal and stress conditions, with their specific function remaining unclear. All detected cytokinin forms (mT, pT, mTR, and pTR) can undergo O-glucosylation, the most prevalent modification in aromatic cytokinins. These O-glucosyl conjugates serve crucial roles in storage, transport, and protection against degradation by enzymes. Specific β-glucosidases readily convert these conjugates back to their active cytokinin forms [21].

Bajguz and Piotrowska’s [21] review highlighted that cytokinin N-glucoside isomers tend to accumulate at higher concentrations compared to their free-base counterparts. In *T. grandis* and *H. guyacan*, the predominant glucoside isomer was N9-glucoside (mT9G). We observed an increase in N-glucosides toward the base of the plant, which is consistent with the findings of Bairu et al. [22], who detected higher levels of N-glucosides at the base of the plant. This is in contrast to many other species, where mT preferentially undergoes glycosylation at the hydroxyl group, forming mTOG [23,24,25,26,27]. This preferential formation of mTOG has been proposed as a factor contributing to its superiority over BA. 

In *T. rosea*, O-glucoside concentrations were significantly higher than those of N-glucosides, particularly within the callus and basal node. oTOG followed an increasing gradient toward the base of the plant, reaching impressive values of 74 and 100% in stems and leaves of the base of the plant. In the callus, it also represented 52% from all ARCKs conjugates. Similarly, *H. guayacan* exhibited elevated oTOG levels throughout the plant, presenting percentages varying between 7–22% in leaves and 13–31% in stems, with the exception of the callus. These findings contrast with those of Montalban et al. [27], who reported only trace amounts of oTOG in *Pinus radiata*, and the complete absence of the isomer in *T. grandis*. Consistent with previous findings, oTROG was not observed in any of the species examined [25,26,27]. A noticeable accumulation of mTOG occurred in *H. guayacan*, with greater concentrations found in leaves compared to stems. This isomer was present in *T. rosea* at lower levels and was entirely absent from *T. grandis*. Mala et al. [26] detected somewhat higher mTOG concentrations in *Ulmus glabra*, while Moyo et al. [27] identified mTOG as a major metabolite of mT in *Amelanchier alnifolia*. Within the callus tissue, mTROG exhibited high concentrations, reaching 32%, 21%, and 69% in the callus of *H. guayacan*, *T. rosea*, and *T. grandis*, respectively. Comparable levels of mTROG were discovered in *Ulmus glabra* [26]. In leaves and stems of *T. rosea*, the mTROG concentration displayed an increasing gradient toward the base of the plant, aligning with the results in *T. grandis*. However, in the leaves and stems of *H. guayacan*, mTROG represented only 1 to 2%.

Our findings demonstrate the presence of pT in both *T. rosea* and *H. guayacan*. While less common, this aligns with its detection in various other species, including *Amelanchier alnifolia* [27], *Pistacia vera* [28], and *Pinus radiata* [25]. This observation supports the hypothesis that pT may serve as a mechanism for plants to deactivate meta-topolin cytokinins, given its lack of demonstrable cytokinin activity [29]. Interestingly, studies in other plant species, such as *Musa* [30] and *Ulmus* [26], did not report pT formation after exogenous mT application. In *H. guayacan* shoots, pTOG concentrations ranged from 7–19% in stems and 9–12% in leaves. It was also detected in *T. grandis* callus, comprising 17% of total ARCK conjugates. Conversely, *T. rosea* primarily exhibited the para topolin isomer in the form of pT9G. The levels of pT9G were highest near the shoot apex, ranging from 3–5% in stems and 5–11% in leaves, while representing only 3% in callus.

Contrary to Abdouli et al. [28] who suggested that the callus limits the exogenous cytokinin uptake in pistachio plants, our results show higher cytokinin concentrations in nodes compared with the callus in both *H. guayacan* and *T. grandis*.

An analysis reveals that the presence and abundance of a particular metabolite vary significantly across the stem, leaves, and callus of different plant species. Two species, *H. guayacan* and *T. rosea*, share a common set of metabolites with the notable exceptions of pTOG (unique to *H. guayacan*) and pT9G (unique to *T. rosea*). Furthermore, metabolite concentrations are markedly higher in *H. guayacan* compared to *T. rosea*. *T. grandis* demonstrated limited O-glucosylation, with mTROG found throughout the plant and pTOG restricted to the callus. None of the investigated species contained oT, oTR, or oTROG. The levels of oT9G were extremely low, while oTOG was present in significant amounts, within the shoots and stems of both *T. rosea* and *H. guayacan*. It is notably abundant in *T. rosea* callus but entirely absent in *H. guayacan* callus. The results suggest that, in *H. guayacan*, oTOG is derived from mTOG or pTOG. In *T. rosea*, only mTOG would be a candidate precursor, but it is only present in very low concentrations. It remains possible that oT, although not detected, could be a precursor that was fully consumed in the reaction. Interestingly, pT derivatives are absent in *T. grandis*, except low amounts within the callus. In general, the data indicate that callus tissue consistently accumulates lower quantities of specific metabolites compared to shoots and leaves.

## 4. Materials and Methods

Explants of 1.5 cm were taken from in vitro donor shoots of *H. guayacan*, *T. rosea*, and *T. grandis*. Stock cultures were produced in 350 mL glass vessels containing modified Murashige and Skoog medium. Ref. [31] with 1/2 NH_4_NO_3_ and 1/2 KNO_3_, supplemented with 30 g/L sucrose and 7 g/L plant agar (Duchefa Biochemie, Haarlem, The Netherlands). The pH was adjusted to 5.8 before autoclaving. Eighteen explants were transferred from stock culture into (145/25 mm) test tubes containing 8 mL of the same basal medium supplemented with 5 µM N^15^ mTR (Olchemim, Olomouc, Czech Republic). After 8 weeks, the leaves and nodes were separated, and samples were lyophilized and sent for hormone analysis. All cultures were maintained in a growth chamber at 26 ± 2 °C under a 16 h photoperiod light provided by PHILIPS master TLD 36 W 830 Reflex ECO (40 μmol m^−2^ S^−1^ PAR). 

Cytokinin analysis was performed as described by Grira et al. [32]: 25–300 mg of lyophilized plant material was homogenized and sonicated, with overnight extraction at −20 °C. For the identification of labeled metabolites of N^15^ mTR, the following internal tracers were added during extraction for recovery purposes: 30 pmol ([^2^H_7_]N6-benzyladenine (D-BA), [^2^H_7_]N6-benzyladenosine (D-BAR), and [^2^H_7_]N6-benzyladenine-9-glucoside (D-BA9G), Olchemim, Olomouc, Czech Republic), taking into account the relative ionization response between topolin and BA derivatives. These extracts were treated and analyzed using the same conditions as described by Grira et al. [32]. The specific N^15^ metabolites were identified using product ion scan mode for selection of the compound-specific transitions for defining the MRM transitions used under MRM mode for the final quantifications. For confirmation, O-glucosides and O-glucoside ribosides were confirmed after *β*-glucosidase treatment and the quantification of the corresponding aglycons. 

## 5. Conclusions

In this study, N^15^ labeled mTR was used to elucidate the intricate pathways used by the three plant species to maintain cytokinin homeostasis. The data revealed N9-glucosylation as the primary mechanism for mTR inactivation, with mT9G identified as the predominant inactive metabolite. The presence of para and ortho derivatives in both *T. rosea* and *H. guayacan* suggests an intriguing metabolic capability: the ability to shift the hydroxyl group from the meta position to both the ortho and para positions on the cytokinin molecule. However, the exact pathway, particularly for the ortho derivatives, remains unclear. Further investigation of the specific enzymatic activities involved in mTR metabolism is necessary to fully understand the ARCKs pathway in plants grown in vitro. In conclusion, our results highlight the remarkable interfamilial and interspecies variation in mTR metabolism, challenging conventional notions such as the role of callus tissue as a primary storage site for cytokinin metabolites.

## Data Availability

Data are contained within the article.
